# Systematic Validation of Protein Force Fields against Experimental Data

**DOI:** 10.1371/journal.pone.0032131

**Published:** 2012-02-22

**Authors:** Kresten Lindorff-Larsen, Paul Maragakis, Stefano Piana, Michael P. Eastwood, Ron O. Dror, David E. Shaw

**Affiliations:** 1 D. E. Shaw Research, New York, New York, United States of America; 2 Center for Computational Biology and Bioinformatics, Columbia University, New York, New York, United States of America; Swiss Federal Institute of Technology Zurich, Switzerland

## Abstract

Molecular dynamics simulations provide a vehicle for capturing the structures, motions, and interactions of biological macromolecules in full atomic detail. The accuracy of such simulations, however, is critically dependent on the force field—the mathematical model used to approximate the atomic-level forces acting on the simulated molecular system. Here we present a systematic and extensive evaluation of eight different protein force fields based on comparisons of experimental data with molecular dynamics simulations that reach a previously inaccessible timescale. First, through extensive comparisons with experimental NMR data, we examined the force fields' abilities to describe the structure and fluctuations of folded proteins. Second, we quantified potential biases towards different secondary structure types by comparing experimental and simulation data for small peptides that preferentially populate either helical or sheet-like structures. Third, we tested the force fields' abilities to fold two small proteins—one α-helical, the other with β-sheet structure. The results suggest that force fields have improved over time, and that the most recent versions, while not perfect, provide an accurate description of many structural and dynamical properties of proteins.

## Introduction

Historically, two principal factors have limited the utility of molecular dynamics (MD) simulations as a research tool in biology and biophysics [1]. First, lengthy and computationally intensive simulations may be required to sufficiently sample the conformational space of the molecules under study. Thanks to recent developments in computing hardware [2]–[4] and in methods to distribute [5] and parallelize [6] simulations or enhance sampling efficiency [7], it is now possible to simulate directly protein dynamics on the millisecond timescale [8] or to reconstruct long-timescale behavior from shorter simulations [9].

Second, in order for MD simulations to provide a realistic description of the molecules under study, the molecular mechanics force field used in such simulations must be sufficiently accurate to provide biologically useful results. A large number of force fields are available for studying proteins by MD simulation. While the mathematical functional forms of many of these force fields are quite similar, they differ in the parameters that describe the various energetic components and in the methods employed to obtain these parameters. Current protein force fields were for the most part derived by fitting parameters to data from quantum-level calculations or experiments on small molecules thought to mimic the properties of proteins. Although most such parameters have not been changed for some time, a number of force fields have recently been refined in order to improve their accuracy for proteins and peptides. These developments have led to a plethora of new force fields that often differ only in the parameters associated with a few (important) torsion angles.

With some notable exceptions [10], [11], previous comparisons of different force fields' abilities to reproduce the structure and dynamics of peptides and proteins have mostly involved only a few versions of related force fields. In most cases such studies have utilized only one or a few related test systems, leaving unresolved questions about how different force fields compare more generally in their ability to provide an accurate description of protein conformational ensembles. Here we describe the results of an extensive test of eight protein force fields, using simulations of a number of diverse protein and peptide systems and subsequent comparison of the resulting conformational ensembles with experimental data.

When evaluating a force field, it is important to sample the test systems as extensively as possible in order to ensure that the level of agreement with experiments is a reliable measure of the accuracy of the force field. The choice of test systems should thus be based in part on the extent to which such systems could be sampled using available computational resources. We have recently described the construction and initial applications of a specialized computer for MD simulations called Anton [2], [8]. Anton is capable of performing simulations that are two orders of magnitude longer than the prior state of the art, enabling much more comprehensive testing of force fields than was previously feasible.

We have used Anton to perform a broad range of computationally demanding tests using protein and peptide systems. For each of the eight different force fields that we examined, we ran a total of 100 µs of simulation distributed across six different molecular systems: (i) two folded proteins, (ii) two peptides that preferentially populate a helical or strand-like structure, respectively, and (iii) an α-helical and a β-sheet protein, simulated at a temperature where they would be expected to fold and unfold. Our results suggest that force fields are improving over time and that, for the tests described here, simulations in two of the force fields result in particularly good overall agreement with experimental data. Our results also highlight certain remaining deficiencies in all force fields studied here and point towards areas for future improvements.

## Results

In the last few years, a substantial number of studies have revised existing force fields by modifying the torsion potentials associated with a few important dihedral angles. Simmerling and colleagues [12] modified the backbone potential in the original Amber ff99 force field by fitting to additional quantum-level data and thus derived the improved Amber ff99SB force field. Best and colleagues followed up on this work by modifying the backbone potential in ff99SB and ff03 to obtain a better energetic balance between helix and coil conformations, thus producing the ff99SB* and ff03* force fields [13]. We modified the side-chain torsion potential for four amino acid types in ff99SB to produce the ff99SB-ILDN force field [14], and more recently we changed parameters associated with both the backbone and certain side chains in a CHARMM force field to produce CHARMM22* [15]. We also demonstrated that the “ILDN” side chain modifications can be combined with the ff99SB* potential to produce the ff99SB*-ILDN force field [15].

We decided to evaluate a number of the modified force fields described above, as well as the force fields from which they were originally derived. We also included the widely used OPLS-AA force field, such that our comparison set was comprised of the following eight protein force fields: Amber ff99SB-ILDN [12], [14], Amber ff99SB*-ILDN [12]–[14] Amber ff03 [16], Amber ff03* [13], [16], OPLS-AA [17], CHARMM22 [18], CHARMM22 with the CMAP correction ([18], [19]; herein termed CHARMM27), and CHARMM22* [15], [18], [19].

### Comparison of simulations with NMR data for folded proteins

MD simulations are often used to study the structural dynamics of folded proteins, and we thus first examined the ability of the eight force fields to reproduce experimental data describing the folded-state structure and dynamics of two well-characterized proteins, ubiquitin and GB3. Both proteins are relatively small (76 and 56 residues, respectively) and have been characterized extensively by solution-state NMR spectroscopy, thus providing experimental data for evaluating the ability of a force field to describe the folded state of a protein correctly [11]. Further, these NMR experiments have shown that the two proteins are very stable and display only relatively little (albeit possibly biologically important) motion on timescales beyond microseconds [20], [21], suggesting that it should be possible to sample the native state ensemble sufficiently well using MD simulations on Anton. We thus performed 10-µs simulations for both ubiquitin and GB3 in each of the eight force fields considered here.

**Figure 1:**
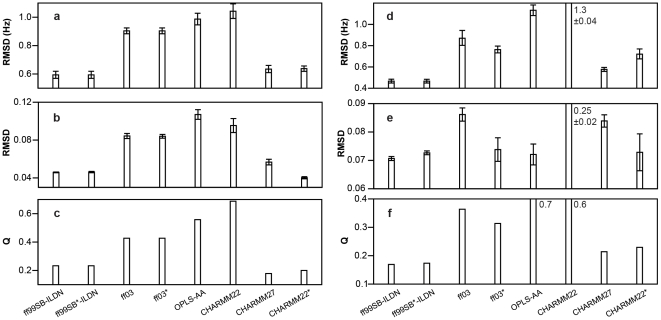
Comparison between simulation and experimental NMR data probing the structure and dynamics of the backbone in folded proteins. The plot shows the results for (a, b, c) ubiquitin and (d, e, f) GB3. In (a, d) we show the agreement between calculated and experimental scalar couplings, in (b, e) the agreement between calculated and experimental order parameters, and in (c, f) the agreement between calculated and experimental residual dipolar couplings. Low RMSD values or Q scores [39] imply better agreement with experiments. Error bars represent the standard error of the mean.

In all but one case we found the native state to be stable and the protein to remain close to the experimental structure throughout the 10-µs MD simulation. (In the case of GB3 simulated with CHARMM22, we found that the native state was not stable and that the protein unfolded during the simulation.) We focused our comparison on the polypeptide backbone because this is the most fundamental part of the protein structure, and because most problems in the description of side chains will eventually reveal themselves at the level of the backbone as the protein distorts in an attempt to accommodate inaccurate rotamer distributions of the side chains. In Figure 1, we show the agreement between the ensembles obtained from simulations and NMR measurements of backbone scalar and residual dipolar couplings as well as NMR order parameters. Overall, four force fields (ff99SB-ILDN, ff99SB*-ILDN, CHARMM27 and CHARMM22*) provide a reasonably accurate description of the native state of ubiquitin and GB3, close to that of ensembles that were reconstructed to fit the experimental data [22]. For the four of the eight force fields that had been studied previously using similar tests [11], we find good agreement between our results and these earlier studies.

### Temperature-dependent structural propensities in short peptides

The simulations of ubiquitin and GB3 provide a detailed test of a force field's ability to describe a well-defined folded state and the fluctuations within that state. In those simulations, only a few substantial conformational excursions are observed. These tests might thus miss differences in force fields that arise, for example, from variations in the relative energies between the different basins on the Ramachandran map. Indeed, although our tests of ubiquitin and GB3 suggested that CHARMM27 and ff99SB-ILDN perform equally well, it has been shown that CHARMM27 severely overstabilizes the formation of helical structures [10], [23] and that ff99SB-ILDN underestimates the stability of helices [13].

**Figure 2:**
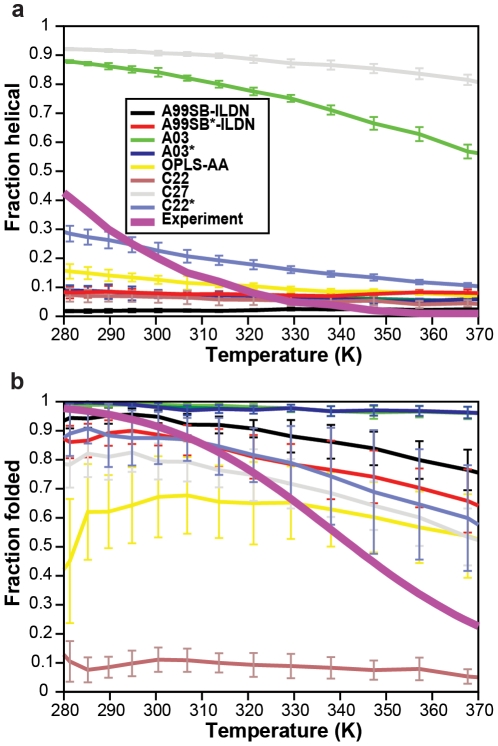
Comparison between calculated and experimental secondary structure propensities. In (a), we show the helical fraction of the (AAQAA)_3_ 15-mer peptide in simulations and experiment as a function of temperature. In (b), we show the fraction folded of the CLN025 10-residue peptide in simulations and experiments as a function of temperature.

We thus performed simulations of two small peptide systems with the aim of evaluating how well the eight force fields provide a balance between propensity to form helical, sheet-like, and coil structures. The first test involves a 15-residue peptide consisting of three repeats of the amino acid sequence AAQAA [24]. NMR and circular dichroism measurements suggest that this peptide is ∼45% helical at a temperature of 275 K, and that the helicity has a relatively steep temperature dependency resulting in less than 10% helicity at 320 K. Using 10 µs of simulated tempering MD simulations, we calculated the temperature-dependent fraction of helical structure of the AAQAA peptide in each of the eight force fields (Fig. 2a). The results confirm that the force fields display a broad range of propensities towards forming helical structure, with CHARMM27 and Amber ff03 overstabilizing helices and ff99SB-ILDN understabilizing them. The three “helix coil–balanced” force fields (ff99SB*-ILDN, ff03* and CHARMM22*) all provide a better description of this peptide system. This result is not surprising, however, given that the comparison with the helicity of the AAQAA peptide was part of the optimization procedure used to refine these force fields.

As a second test, we performed a comparable set of calculations for the 10-residue peptide CLN025 [25]. CLN025 preferentially attains a hairpin-like structure in solution at temperatures less than ∼340 K, and only at higher temperatures is the unfolded “coil” structure the free-energy minimum. In Figure 2b, we show the temperature-dependent stability of CLN025 in the eight force fields and the comparison to the experimentally derived melting curve. As for the AAQAA system, the eight force fields display a broad range of behaviors, with the very helical CHARMM27 force field being the biggest outlier, as it forms almost no folded structures at any temperature.

In addition to quantifying the different force fields' ability to capture the subtle balance between helical, sheet-like and coil structures, these simulations also highlight an important deficiency in current molecular mechanics force fields [13]. In particular, even for the most well-balanced force fields, the temperature dependency of the melting of the AAQAA-helix and the native state of CLN025 is much weaker than suggested from experiment. This in turn means that these force fields can match the experiments closely only in a narrow range of temperatures. Capturing the cooperativity of helix and hairpin formation and melting thus appears to be a general area for further improvement of force fields.

### Simulating the folding of α-helical and β-sheet proteins

The ability to fold a protein from an unfolded state to the correct native structure is a very stringent test of a molecular mechanics force field [26]. For many proteins, the folding free energy is relatively small, suggesting that even minor force field errors could result in the native state not being the free-energy minimum in simulation. Further, the folding process from an unfolded to a folded state involves structural changes throughout the protein—at the level of both the side chains and the polypeptide backbone—causing small errors in individual force field terms (such as for the backbone torsions) to be amplified [27]. As a result, simulations of protein folding might be able to detect even relatively minor problems in force fields. In certain cases, it might be possible to compensate for such force field deficiencies in folding simulations by choosing a force field that overstabilizes the secondary structure found in the native state. A more stringent test of a force field is thus to be able to find the native state of at least two proteins of very different structural classes, such as one with an α-helical structure and one with a β-sheet [8], [28].

The timescales for the folding of even the fastest-folding proteins are in the microsecond range, so systematic studies of different force fields' abilities to fold proteins have previously been computationally demanding. Anton's ability to perform long MD simulations now makes such a test feasible, and we performed folding simulations for two proteins using all eight force fields. For these tests, we chose the fastest folding α- and β-proteins known: the Nle/Nle double mutant of the villin headpiece, a small α-helical protein with a folding time of ∼1 µs [29]; and the GTT variant of the FiP35 WW domain, which folds in ∼4–6 µs [30] and whose native state consists of three β-strands.

Protein folding is a stochastic process, and one expects considerable variation in the exponentially distributed waiting times between individual folding events. For both villin and the WW domain, we performed simulations with a length ∼10 times the experimentally determined folding time: simulation lengths were 10 µs for villin and 50 µs for the WW domain. We performed these simulations at the experimental melting temperature, where the experimental folding and unfolding times are equal. Simulations with an accurate force field that are an order of magnitude longer than the experimental average folding time are expected to result in the observation of reversible folding and unfolding.

Table 1 shows the number of folding and unfolding events observed during the simulations of villin and WW in each of the eight force fields. In six of the eight force fields we were able to fold villin to its correct native state, and in five force fields the WW domain folded. In four force fields we were able to fold both villin and the WW domain; these include all three helix-coil balanced force fields (ff99SB*-ILDN, ff03* and CHARMM22*) as well as ff99SB-ILDN (in agreement with earlier findings [8]). The two very helical force fields (ff03 and CHARMM27) were able to fold villin correctly, but were not able to fold the β-sheet–containing WW domain. For both of these force fields the villin domain folded relatively quickly (0.2 µs and 0.8 µs for ff03 and CHARMM27, respectively), after which the protein stayed folded for the remainder of the simulation, suggesting that the native state is too stable, as also observed previously [15]. For all four force fields that were able to fold both proteins, we also observed reversible folding and unfolding of villin; only for CHARMM22* did we observe reversible folding of both proteins.

**Table 1:** Evaluating force fields by folding simulations.

Force field	Villin	WW
Amber ff99SB-ILDN	**✓** (1/1)	**✓** (1/0)
Amber ff99SB*-ILDN	**✓** (2/2)	**✓** (1/0)
Amber ff03	**✓** (1/0)	**✗**
Amber ff03*	**✓** (1/1)	**✓** (1/1)
OPLS-AA	**✗**	**✓** (1/0)
CHARMM22	**✗**	**✗**
CHARMM27	**✓** (1/0)	**✗**
CHARMM22*	**✓** (4/4)	**✓** (1/1)

The table shows whether we observed any folding events of villin and the WW domain in our simulations. A check mark indicates that simulations started in the unfolded state were able to reach the folded state in 10 µs (villin at 360 K) or 50 µs (WW domain at 370 K), while an “X” means that we did not observe any folding events. In those cases where we did observe at least one folding event, the numbers in parentheses indicate the number of folding/unfolding events we observed in that simulation. For ff99SB-ILDN, for example, we observed first a folding event and subsequently an unfolding event for villin, but only a folding event with no subsequent unfolding event for the WW domain. Since the simulations are roughly 10 times longer than the experimental folding and unfolding times, one would expect roughly five folding and five unfolding events in a force field that models perfectly both the kinetics and thermodynamics of folding.

Even minor force field deficiencies can result in substantial changes in calculated folding rates and melting temperature—even in the case where the folded state is a free-energy minimum. Because the simulations were run for only 10 times the experimental folding time and only at a single temperature, one should avoid overinterpreting the results in Table 1. Nevertheless, it is worth noting that the three force fields that were parameterized to obtain a reasonable balance between helical and coil structures were all able to fold both an α-helical and a β-sheet protein. The agreement between these two different tests suggests that the smaller (and easier to sample) peptide systems indeed contain useful information that can be used to optimize force fields for application to conformational changes in proteins. As sampling efficiency and force fields continue to improve in the future, we expect that more detailed and quantitative studies of the thermodynamics and kinetics of folding [31] might provide even more stringent tests of force fields.

## Discussion

We have presented a systematic comparison of a number of force fields for all-atom simulations in explicit solvent. Although several of the test systems have previously been used individually to evaluate force fields, the broad nature of the tests applied here—as well as the increased length of the simulations—allows us to draw broader conclusions about the ability of the various force fields to reproduce a range of experimentally measured properties correctly. For example, while Amber ff99SB-ILDN and CHARMM27 appear to describe folded proteins equally well (Fig. 1), our tests on flexible peptides (Fig. 2) revealed large differences between these two force fields. We thus stress the need for validating force fields using as broad a set of systems as possible.

**Figure 3:**
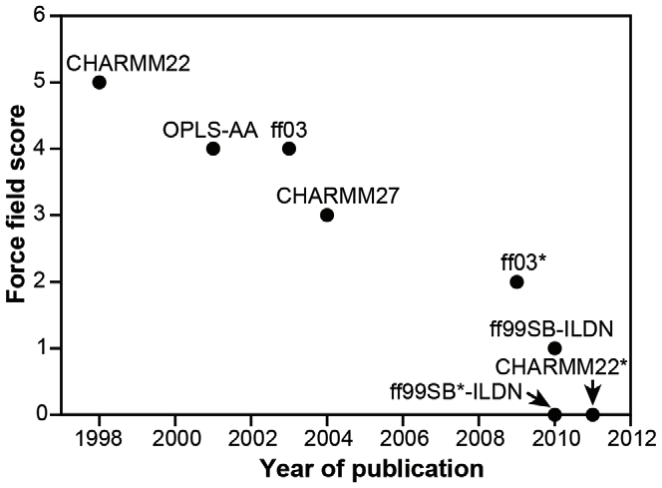
Improvement of force fields over time. For each force field, we assigned a score depending on the agreement with experiments in the tests presented here. Low scores indicate good agreement with experiments. These scores are plotted against the year in which the force field was published. For the force fields that involve multiple corrections (e.g., ff99SB*-ILDN), we use the year of the most recently published correction.

In an attempt to evaluate and compare the different force fields across all three sets of tests, we assigned a “force field score” reflecting the degree of agreement between the experiments and simulations. While one might in principle define a score directly from a quantitative comparison between the calculated and experimental results, such a score would depend on a number of somewhat arbitrary parameters and functional forms used to aggregate across the different results. Instead, we decided to assign a very simple score manually (using integer values ranging from 0 to 6, with low values indicating good agreement with experiments; see Methods for details), thus explicitly acknowledging that the score relies in part on subjective choices and that others might assign different scores based on the same set of results. The assigned scores indicate that two of the eight force fields, ff99SB*-ILDN and CHARMM22*, perform consistently well in reproducing the experimental data in the set of tests presented here (Fig. 3). As these two force fields are also among the most recent ones, we examined whether force fields have generally improved over time. The results show a clear correlation between the year of publication of a force field and the assigned force field score, suggesting that force fields are indeed improving over time (Fig. 3).

It should be noted that other factors will often be relevant to the choice of a force field for specific types of MD simulations. Such factors may include the availability of force field parameters for molecules other than proteins (e.g., lipids, nucleic acids, carbohydrates, co-factors, substrates or drug molecules). In particular, it should be noted that our tests do not include any membrane proteins, and that the force field best used to describe such proteins might in principle depend on the lipid model employed.

Our results also highlight areas for future improvements of the force fields we tested. These include the ability to model the temperature dependency of the conformational propensities in both the AAQAA and CLN025 peptides, and to more accurately match the kinetics and thermodynamics of the folding of villin and the WW domain. We are hopeful that the tests described here will prove useful in further refining contemporary force fields, thus enhancing the value of MD simulation as a tool for elucidating the molecular details of important biological processes.

## Methods

**Common methods.** All production molecular dynamics simulations were performed on Anton [2]. Simulations were performed in the TIP3P water model ([32]; for Amber and OPLS-AA force fields) or the CHARMM modified TIP3P water model ([18], [32]; for CHARMM force fields).

**Simulations and analysis of the native state of ubiquitin and GB3.** Production simulations of ubiquitin and GB3 were performed in the NVT ensemble. We used a 9.5-Å cutoff for the Lennard-Jones and short-range electrostatic interactions; long-range electrostatic interactions were treated with the Gaussian split Ewald method [33]. The starting structures for the simulations were the high-resolution NMR structures of ubiquitin ([34]; PDB entry 1D3Z) and GB3 ([35]; PDB entry 1P7E). The structures were solvated in a cubic box with side lengths 58 Å, and were first minimized, heated to 300 K during 0.4 ns, and finally equilibrated in the NPT ensemble for 0.8 ns. The frame with the volume closest to the average during this NPT simulation was used as starting point for the production simulations in the NVT ensemble, thus ensuring that the average pressure in the simulations is close to the reference standard pressure. For both ubiquitin and GB3 we also performed simulations in the NPT ensemble (using ff99SB*-ILDN) and found that the calculated NMR observables are within error the same as those in the corresponding simulations in the NVT ensemble.

We calculated backbone scalar couplings using published Karplus relationships for HNHA, HNCO and HNCB [36], and HACO [37] couplings and compared to experimental data measured for ubiquitin ([38]; HNHA, HNCO, HNCB and HACO) and GB3 ([36]; HNHA, HNCO and HNCB). We calculated backbone residual dipolar couplings and the associated Q scores as previously described [39] and compared to experimental values in ubiquitin [34] and GB3 [35]. Order parameters were calculated from the values of the internal autocorrelation functions at lag times close to the experimentally determined rotational correlation times.

**Simulated tempering simulations and analysis of AAQAA and CLN025 peptides.** The temperature-dependent conformational properties of the (AAQAA)_3_ [24] and CLN025 [25] peptides were obtained using simulated tempering simulations [40] in the NPT ensemble. In contrast to the simulations of folded proteins or of protein folding, we found it necessary to perform these simulations in the NPT ensemble to avoid changing the average pressure as the temperature varied. We used a 9.5-Å cutoff for the Lennard-Jones and short-range electrostatic interactions; long-range electrostatic interactions were treated with the Gaussian split Ewald method [33].

The helical fraction of the AAQAA-peptide was calculated as the fraction of helical residues [13], [15] at each temperature in the simulated tempering simulations and compared to the experimental values [24]. The fraction of the CLN025 that was folded was determined by applying a dual-cutoff approach [15], [41] to separate the simulations into folded and unfolded states. In this analysis, a folding event was recorded if the Cα-RMSD to the experimental NMR structure dropped below 1.0 Å and an unfolding event was recorded once the same RMSD went above 4.0 Å.

**Folding simulations of villin and WW domain.** Simulations of fast-folding variants of villin [29] and the WW domain [30] were performed in the NVT ensemble using a Nose-Hoover thermostat and a force-shifted cutoff [42] of 10.0 Å (villin) or 10.5 Å (WW domain) for the Lennard-Jones and electrostatic interactions. The starting structures for the simulations were heat-unfolded states of the two proteins in a cubic box of water with side length 52 Å. The simulations were performed near the experimental melting temperatures (at 360 K for villin and 370 K for the WW domain). For the WW domain, we recorded a folding event when the Cα-RMSDs (to PDB entry 2F21) calculated over four stretches of amino acids all were below the cutoff value: 2–33 (2.0 Å), 8–22 (1.1 Å), 12–18 (0.6 Å), 19–30 (0.9 Å). An unfolding event was recorded when the same set of RMSDs went above 7.0 Å, 5.8 Å, 1.8 Å and 3.8 Å, respectively. For villin, we recorded a folding event when the Cα-RMSDs (to PDB entry 2F4K) calculated over three stretches of amino acids were all below the cutoff value: 3–31 (1.2 Å), 3–18 (0.9 Å), 14–31 (0.9 Å). An unfolding event was recorded when the same set of RMSDs simultaneously went above 5.0 Å, 4.6 Å, and 2.5 Å, respectively.

**Assigning a force field score.** For each of the three sets of tests we manually assigned to each force field a number in the range 0–2, with 0 referring to a reasonable agreement, 1 to some agreement and 2 to severe discrepancies with respect to the experimental data. The assigned scores for each of these tests (folded proteins/peptides/folding) were 0/1/0 (ff99SB-ILDN), 0/0/0 (ff99SB*-ILDN), 1/2/1 (ff03), 1/1/0 (ff03*), 2/1/1 (OPLS-AA), 2/1/2 (CHARMM22), 0/2/1 (CHARMM27) and 0/0/0 (CHARMM22*). Each force field was then assigned an overall score (between 0 and 6) that was the sum of the values for each of the three tests. When evaluating the results of the simulations of the AAQAA and CLN025 peptides, we focused mostly on the temperature range around 280–320 K, where most biomolecular simulations are performed. Since simulations of the AAQAA peptide were used in the re-parameterization of the three helix coil–balanced force fields, one could argue that these results should not be included in the evaluation. The nature of the results presented in Figure 3, however, would not change even if the AAQAA tests were excluded. Finally, we stress that the assigned scores rely in part on subjective choices and that different sets of scores could be derived from the data presented in Figures 1 and 2 and Table 1.
