# Correction: Systematic Validation of Protein Force Fields against Experimental Data

**DOI:** 10.1371/annotation/8301b5d4-1ba3-40e7-8fcd-3e169b967044

**Published:** 2013-04-26

**Authors:** Kresten Lindorff-Larsen, Paul Maragakis, Stefano Piana, Michael P. Eastwood, Ron O. Dror, David E. Shaw

There was an error in Figure 1. The correct version of Figure 1 is available here: 

**Figure:**
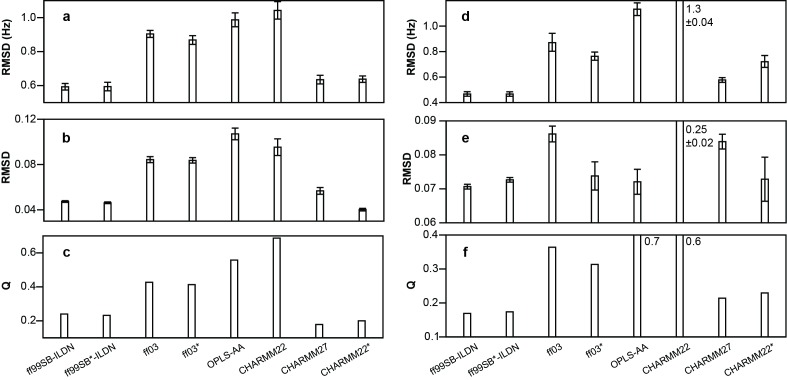


There were errors in the last sentence of the third paragraph of the section "Temperature-dependent structural propensities in short peptides." The correct sentence is:

As for the AAQAA system, the eight force fields display a broad range of behaviors, with the CHARMM22 force field being the biggest outlier, as it forms almost no folded structures at any temperature. 

